# Novel concepts and improvisation for treating postpartum haemorrhage: a narrative review of emerging techniques

**DOI:** 10.1186/s12978-023-01657-1

**Published:** 2023-08-11

**Authors:** G. J. Hofmeyr

**Affiliations:** 1 Department of Obstetrics and Gynaecology, University of Botswana, Notwane Rd, Gaborone, Botswana; 2 Universities of the Witwatersrand and Walter Sisulu, East London, South Africa

**Keywords:** Postpartum haemorrhage, Novel treatments, Improvisation, Ingenuity, Evidence

## Abstract

**Background:**

Most treatments for postpartum haemorrhage (PPH) lack evidence of effectiveness. New innovations are ubiquitous but have not been synthesized for ready access.

**Narrative review:**

Pubmed 2020 to 2021 was searched on ‘postpartum haemorrhage treatment’, and novel reports among 755 citations were catalogued. New health care strategies included early diagnosis with a bundled first response and home-based treatment of PPH. A calibrated postpartum blood monitoring tray has been described. Oxytocin is more effective than misoprostol; addition of misoprostol to oxytocin does not improve treatment. Heat stable carbetocin has not been assessed for treatment. A thermostable microneedle oxytocin patch has been developed. Intravenous tranexamic acid reduces mortality but deaths have been reported from inadvertent intrathecal injection. New transvaginal uterine artery clamps have been described. Novel approaches to uterine balloon tamponade include improvised and purpose-designed free-flow (as opposed to fixed volume) devices and vaginal balloon tamponade. Uterine suction tamponade methods include purpose-designed and improvised devices. Restrictive fluid resuscitation, massive transfusion protocols, fibrinogen use, early cryopreciptate transfusion and point-of-care viscoelastic haemostatic assay-guided blood product transfusion have been reported. Pelvic artery embolization and endovascular balloon occlusion of the aorta and pelvic arteries are used where available. External aortic compression and direct compression of the aorta during laparotomy or aortic clamping (such as with the Paily clamp) are alternatives. Transvaginal haemostatic ligation and compression sutures, placental site sutures and a variety of novel compression sutures have been reported. These include Esike’s technique, three vertical compression sutures, vertical plus horizontal compression sutures, parallel loop binding compression sutures, uterine isthmus vertical compression sutures, isthmic circumferential suture, circumferential compression sutures with intrauterine balloon, King’s combined uterine suture and removable retropubic uterine compression suture. Innovative measures for placenta accreta spectrum include a lower uterine folding suture, a modified cervical inversion technique, bilateral uterine artery ligation with myometrial excision of the adherent placenta and cervico-isthmic sutures or a T-shaped lower segment repair. Technological advances include cell salvage, high frequency focussed ultrasound for placenta increta and extra-corporeal membrane oxygenation.

**Conclusions:**

Knowledge of innovative methods can equip clinicians with last-resort options when faced with haemorrhage unresponsive to conventional methods.

## Plain language summary

Unborn babies are nourished by the mother’s blood supply to the afterbirth (placenta). After a baby’s birth, contractions of the muscles of the womb (uterus) shear the placenta from its attachment and clamp off the mother’s blood vessels. This process is assisted by giving an injection which contracts the womb, and pulling gently on the cord to remove the afterbirth. Usually, blood loss is minimal. Occasionally the womb fails to contract effectively or there is bleeding from tears of the birth canal. Bleeding in excess of 500 ml is called postpartum haemorrhage, which can be life-threatening. Excessive bleeding may be treated with medicines to contract the womb or help the blood to clot, rubbing or squeezing the womb, a ‘drip’ with fluids or blood replacement, placing a balloon or suction device in the womb, and operations to stop the bleeding or remove of the womb (hysterectomy). There is very little hard evidence for the effectiveness of each method. This review highlights recent innovations including approaches to quick diagnosis and rapid treatment, suturing the lower part of the womb via the vagina, improvements in balloon and suction methods, new designs of clamps to apply through the vagina and to clamp the aorta (the main artery of the body), new approaches to giving blood clotting factors, many new types of stitches to compress the uterus, and advanced technology such as ultrasound treatment and artificial oxygenation of the mother’s blood. We call for more research to show which methods really work.

## Background

Few health problems distinguish low-income from well-off communities more starkly than postpartum haemorrhage (PPH). PPH is the most common direct cause of maternal deaths in low-resource settings, where 99% of global deaths from PPH occur, while death from PPH in well-resourced settings is rare. This is not surprising. Bleeding results from breaches in the integrity of the vascular system which should be controllable in most cases, but such control often requires a well-developed health system with resources such as advanced surgical and radiological capability, laboratory services, high-dependency nursing care and blood transfusion.

The International Federation of Gynaecology and Obstetrics (FIGO) has developed recommendations for the treatment of PPH [1] which endorse the 2020 WHO ‘bundle’ approach [2] to first response and refractory PPH. The FIGO recommendations include:

First response bundle:

- Intravenous oxytocin
- isotonic crystalloids
- Early intravenous tranexamic acid
- Uterine massage.

Response to refractory PPH bundle:

- Bimanual uterine compression or external aortic compression
- Uterine balloon tamponade (with call for better evidence)
- Nonpneumatic antishock garment.

In addition:

- Uterine artery embolization if available
- Surgical interventions: compression suture techniques, uterine and hypogastric artery ligation, and hysterectomy.

The document also calls for early diagnosis based on both observed blood loss and clinical signs of blood loss, including a shock index ≥ 0.9; and tools for objective monitoring of blood loss.

## Evidence of effectiveness

It is reasonable to assume that overall, current treatments for PPH are effective, given the continuing decrease in deaths from haemorrhage in well-resourced settings, to levels as low as 1.0 per 100,000 live births [3]. However, most treatment methods for PPH have entered clinical practice with remarkably little evidence of effectiveness.

A Cochrane review of mechanical and surgical interventions for treating primary postpartum haemorrhage found insufficient evidence of effectiveness for any method and called for robust research [4]:

- External uterine compression: unclear (n = 64; risk ratio (RR) for blood transfusion 2.33, 95% confidence interval (CI) 0.66 to 8.23)
- Uterine arterial embolisation versus surgical devascularisation plus B-Lynch: unclear (n = 23; RR for hysterectomy 0.73, 95% CI 0.15 to 3.57)
- Uterine balloon tamponade with condom catheter: unclear (n = 116; RR for blood loss of 1000 ml or more 1.52, 95% CI 1.15 to 2.00; 113 women); mortality due to bleeding RR 6.21, 95% CI 0.77 to 49.98); hysterectomy to control bleeding (RR 4.14, 95% CI 0.48 to 35.93); total blood transfusion (RR 1.49, 95% CI 0.88 to 2.51);
- Latex balloon catheter together with cervical cerclage: unclear (n = 240; hysterectomy RR 0.14, 95% CI 0.01 to 2.74; additional surgical interventions to control bleeding RR 0.20, 95% CI 0.01 to 4.12)
- Bakri balloon tamponade versus haemostatic square suturing of the uterus: unclear (n = 13; blood transfusion RR 0.57, 95% CI 0.14 to 2.36; 'intraoperative' blood loss (mean difference (MD) − 426 ml, 95% CI − 631.28 to − 220.72).
- Bakri balloon versus condom catheter: uncertain (n = 66; hysterectomy RR 0.50, 95% CI 0.05 to 5.25; blood transfusion RR 0.97, 95% CI 0.88 to 1.06).
- Bakri balloon with versus without a traction stitch: unclear (n = 50; hysterectomy RR 0.20, 95% CI 0.01 to 3.97).
- Condom catheter versus gauze packing: uncertain (n = 212; fever RR 0.47, 95% CI 0.38 to 0.59).
- Modified versus standard B-Lynch compression suture: may reduce hysterectomy (n = 160; RR 0.33, 95% CI 0.11 to 0.99) and postoperative blood loss (MD − 244.00 ml, 95% CI − 295.25 to − 192.75).

## Misleading observational impressions

There are at least two reasons for the lack of robust data. Firstly, randomized trials of complex interventions are extremely difficult to conduct. PPH refractory to first-line management is rare, and recruitment to trials in the setting of acute haemorrhage is difficult. Recent developments in the concept of deferred consent have mitigated consent barriers to some extent, but this remains a difficult area [5]. Secondly, while refractory PPH is an acute, life-threatening condition which provokes great anxiety for the caregiver, most patients recover irrespective of the treatment. The result is that clinicians experience an unrealistic impression of the effectiveness of whatever method they use, and become convinced of the effectiveness of their methods, to the extent of regarding randomized trials as unethical.

An instructive example of unrealistic faith in a treatment method is misoprostol. In July 1995 it occurred to the author that misoprostol, an inexpensive, orally active prostaglandin analogue with powerful uterotonic effects should be the ideal treatment for PPH. The concept seemed too self-evident to require confirmation, and was supported by spectacular results from an observational report of 100% arrest of bleeding within 3 min of administering rectal misoprostol for refractory PPH [6]. In contrast with the widespread clinical experience of misoprostol as a life-saving treatment for PPH, network meta-analysis of randomized trials of misoprostol for treatment of PPH found that, compared with oxytocin, misoprostol probably increases the risk of blood transfusion (risk ratio (RR) 1.47, 95% confidence interval (CI) 1.02 to 2.14), and may increase the incidence of additional blood loss of 1000 ml or more (RR 2.57, 95% CI 1.00 to 6.64). The relative effect on maternal death or severe morbidity was unclear (RR 1.98, 95% CI 0.36 to 10.72) [7]. A systematic review of randomized trials of both prevention and treatment of PPH found that 11 of 15 maternal deaths occurred in the misoprostol groups (31 studies; misoprostol 11/19,715 deaths versus placebo or other uterotonics 4/20,076 deaths; risk ratio (RR) 2.08, 95% confidence interval (CI) 0.82 to 5.28). All 11 deaths in the misoprostol arms occurred with misoprostol dosage ≥ 600 µg [8]. In summary, despite its widespread use and overwhelmingly positive clinical experience, there is no robust evidence that adding misoprostol to the usual treatment of PPH reduces blood loss or mortality.

The importance of this example is that other treatment methods with apparently beneficial effects in observational studies and from clinical experience, need confirmation from randomized trials to be sure that they do more good than harm.

## Novel concepts

The large number of innovative treatment methods reported recently is testimony to the inventiveness of clinicians faced with the prospect of loss of life from haemorrhage, and the fact that no single method is effective in all cases. These innovations are spread across a wide range of publications and have not been summarized in a single paper for easy access. To identify novel concepts in the treatment of PPH, the author searched Pubmed 2021 to 2022 on the terms ‘postpartum haemorrhage treatment’ (755 publications, 20 May 2022). The titles and where indicated abstracts and full texts were reviewed. Novel treatments are catalogued below and in Table 1 as a summary of techniques which have recently been described, emphasizing the dearth of evidence for effectiveness of almost all methods.

**Table 1:** Summary of recent approaches to treatment of PPH (see original papers for illustrations of surgical techniques described)

**Home-based treatment**	
Family training program [16]	
**Clinical care protocols**	
Risk assessment, measurement of blood loss, structured escalation and point-of-care viscoelastometric-guided early fibrinogen replacement [11]	
**Obstetric emergency team training**	
Co-ordination of and communication during emergencies [15], PPH kits [18]	
**First line treatment**	
E-MOTIVE: Early detection with calibrated drape plus ‘MOTIVE’ bundle [13]	
**Early diagnosis**	
Reusable calibrated postpartum blood collection tray [14]	
**Appropriate uterotonics** [8]	
**Tranexamic acid**	
Intravenous (not oral [27]) tranexamic acid for PPH treatment [23], not caesarean prophylaxis [25]	
**Non-pneumatic anti-shock garment**	
Recommended by WHO [3]	
**Refractory PPH, non-surgica**l	
Suction uterine tamponade devices [41, 42] versus balloon tamponade modifications [37]	
**Vaginal balloon tamponade** [38]	
**Restrictive fluid resuscitation**	
Permissive hypotension to reduce blood loss [43]	
**Massive transfusion protocols** [44]	
Early fibrinogen [45] and cryoprecipitate [47] not proven	
**Point-of-care viscoelastic haemostatic assay-guided blood product transfusion** [48]	
**Pelvic artery embolization**	
Limited availability [53, 54]	
**Endovascular balloon occlusion of the aorta, common ileac arteries or internal ileac arteries** [56]	
**External aortic compression**	
Recommended by WHO [2]	
**Direct compression of the aorta during laparotom**y	
Digital compression or various aortic clamps [65, 66]	
**Transvaginal haemostatic ligation procedures**	
Various techniques described [67, 68]	
**Placental site sutures**	
Purse string technique [69]	
**Uterine compression sutures**	
Multiple variations described [71–79], including removable suture [80]	
**Innovative measures to control lower uterine segment haemorrhage associated with placenta accreta spectrum**	
Many suture techniques described [81–85]	
**‘Sandwich’ technique**	
B-LYNCH suture plus intrauterine balloon [86]	
**Uterine devascularization**	
Stepwise approach [88]	
**Cell salvage**	
Feasible but complex [90–92]	
**High frequency focussed ultrasound for placenta increta**	
Promising [93]	
**Veno-arterial extra-corporeal membrane oxygenation (VA-ECMO)**	
Oxygenation pending cardiopulmonary recovery [94, 95]	

### Clinical care protocols

National implementation of an ‘Obstetric Bleeding Strategy for Wales’ comprising universal risk assessment, quantitative measurement of blood loss, structured escalation to senior clinicians and point-of-care viscoelastometric-guided early fibrinogen replacement [9, 10], was associated with reduction in massive postpartum haemorrhage and blood transfusion [11].

### Early diagnosis and bundled first response

The "E-MOTIVE" intervention comprising early PPH detection using a calibrated drape, uterine Massage, oxytocic drugs, tranexamic acid, intravenous fluids, genital tract examination and escalation when necessary reduces the composite outcome severe PPH (> 1000 ml) or hysterectomy or death from haemorrhage by 60%.[12, 13].

### Early diagnosis

A reusable calibrated postpartum blood collection tray for blood loss monitoring has been assessed for patient and provider acceptability [14].

### Obstetric emergency team training

In recent years increasing emphasis has been placed on the importance of obstetric emergency team training to strengthen co-ordination of and communication during emergencies, but the effect of such training on severe PPH is unclear [15].

### Home-based treatment of PPH

A novel program training family members to administer misoprostol in the event of haemorrhage after home birth was found to be effective in terms of numbers of women with PPH receiving treatment at home [16].

### Early risk stratification

Early prediction of PPH severity has been used to facilitate the treatment of PPH. Delta neutrophil index (DNI) and the clinical shock index have been found to be acceptable predictors both individually and combined [17].

### Postpartum haemorrhage kits

PPH kits and carts [18] have become standard in many settings, to ensure that all the equipment and consumables are readily available in the event of an emergency. An exception is oxytocin which needs to be refrigerated. Ergometrine has been shown to be stable at room temperature for 6 months provided shielded from light [19].

### Uterotonics

A network meta-analysis found that oxytocin is more effective than misoprostol for first-line treatment of PPH, with fewer adverse side-effects; addition of misoprostol to oxytocin has little or no benefit; and there is little robust evidence on other uterotonics such as ergometrine and injectable prostaglandins [8].

In low-resource settings, manufacturing quality and degradation due to ineffective cold chain management are major obstacles to the efficient use of oxytocin [20]. While 100mcg heat stable carbetocin (HSC) has been shown to have similar effectiveness to 10 units of oxytocin for prevention of PPH [21], we are not aware of evidence of the safety or effectiveness of HSC for treatment.

A thermostable microneedle oxytocin patch has been developed to extend access to oxytocin to lower-level settings [22].

### Intravenous tranexamic acid

Systematic reviews have concluded that early administration of tranexamic acid for treatment of PPH is life-saving and cost-effective [23]. Evidence of effectiveness to reduce blood loss has resulted in its routine use prophylactically at caesarean section [24]. The effectiveness of prophylactic tranexamic acid at caesarean birth has not been confirmed in the largest randomized trial to date [25]. Unfortunately, observational data suggest that the greater availability of tranexamic acid in caesrean section theatres has been associated with an increase in mortality from inadvertent use of tranexamic acid for spinal analgesia [26]. This highlights how difficult it is to predict unanticipated adverse effects of introducing new technologies. The benefits of tranexamic acid can be preserved and the risks eliminated by never storing tranexamic acid inside the operating theatre [26].

### Oral tranexamic acid

A placebo-controlled trial found no improved clinical outcomes with adjunctive use of oral tranexamic acid for treatment of PPH [27].

### Non-pneumatic anti-shock garment

The medical antishock trousers (MAST) suit was originally developed as an inflatable compression suit to counteract G-force-associated reduction in venous return associated with high-speed military aviation. More recently a systematic review of a neoprene non-pneumatic antishock garment in the management of PPH has found a trend to improved outcomes [28], and the method has been recommended by a WHO technical committee as part of the bundle of care for refractory PPH [3].

## Transvaginal uterine artery clamp (TVUAC)

TVUAC is an intuitively attractive method due to its directness, speed and simplicity. The uterine arteries are camped in the lateral vaginal fornices [29]. A clamp with doppler detector to identify the uterine arteries has also been used in the treatment of fibromyomata [30]. Control of bleeding in 153 cases of PPH, of which 33 required additional surgical interventions, using a specially designed clamp has been reported [31]. Traction is applied to the cervix at 12 and 6 o’clock and the clamps are applied to each uterine artery via the lateral vaginal fornix [32]. The author uses a different approach, applying traction to the cervix first at 3 o’clock and clamping the left uterine artery with ring forceps or Green-Armytage forceps with one jaw inside the cervix and one in the lateral fornix, then repeating at 9 o’clock.

## Novel approaches to uterine balloon tamponade

Fixed volume uterine balloon tamponade [1] has become part of routine PPH management, recommended by WHO [2] and most professional associations, based on extensive observational data indicating an 83% to 95% success rate in controlling haemorrhage [33]. The only two randomized trials identified had negative results, and a systematic review concluded that the effect of balloon tamponade devices was unclear [34].

### Improvisation versus purpose-designed devices

A case can be made for the notion that improvised devices, even if not quite as effective as purpose-designed devices, may do more good in the world due to global availability. An example is uterine balloon tamponade, which was made available globally in a short period of time, because balloons could be easily made from a condom and a catheter, as opposed to the time-consuming processes of registration and distribution of purpose-designed devices. The cost of uterine tamponade devices ranges from USD 0.64 to USD 400 [35].

### Free-flow versus fixed volume balloons

Since 2001 the author has empirically used an improvised method of uterine balloon tamponade using a surgical glove as a balloon, and instead of filling with a fixed volume, attaching the balloon to an intravenous administration set to maintain constant pressure as the uterus contracts and relaxes. In collaboration with colleagues from University of Stellenbosch, we described this method, including their novel method of sealing the balloon around the catheter [36]. These concepts were subsequently incorporated into the Elavi free-flow uterine balloon tamponade system [37]. There is to date no robust evidence as to whether the free-flow or the fixed volume approach is more effective.

## Vaginal balloon tamponade

Use of a condom balloon retained with stay sutures between the labia minora to control bleeding from multiple vaginal lacerations has been described [38].

## Uterine suction tamponade

An alternative approach to uterine tamponade is suction tamponade. This is intuitively attractive because it is aligned with the physiological mechanism of postpartum haemostasis, uterine contraction and retraction. Several purpose-designed and improvised suction devices have been used for this purpose. The Jada system (Alydia Health) has been approved by the Federal Drug Administration in the United States of America [39]. Improvised use of a Bakri balloon catheter as a suction catheter is in routine use at the University Hospital, Zurich, Switzerland [40]. Use of an inexpensive Levin stomach tube FG 24 to 36 for ‘suction tube uterine tamponade’ has been reported [41, 42].

## Restrictive fluid resuscitation

The concept of permissive hypotension is relatively recent with respect to PPH management. Large volume intravenous crystalloid infusion may have adverse effects such as hypothermia, acidosis and coagulopathy. A randomized trial found that a restrictive policy (0.75 to 1 × the blood volume loss compared with standard policy (1.5 to 2 × blood volume loss) was associated with a non-significant reduction in risk of blood loss > 1000 ml (− 12%, 95% confidence interval − 24.3% to 0.3%) [43].

## Massive transfusion protocols

Formal protocols for transfusion of multiple blood product units have been suggested to improve outcomes for severe postpartum haemorrhage [44].

## Fibrinogen

A placebo-controlled trial of early, systematic use of fibrinogen concentrate in the treatment of PPH did not demonstrate improved clinical outcomes [45].

## Early cryoprecipitate transfusion

Encouraging results from a pilot study [46] and a retrospective cohort study [47] justify further investigation of this approach.

## Point-of-care viscoelastic haemostatic assay-guided blood product transfusion

In recent years there has been increasing use of point-of-care viscoelastic haemostatic assay-guided blood product transfusion [48]. In an evaluation of a national quality improvement project in Wales including this technology, there were no deaths from haemorrhage among 60,914 maternities [49]. The clinical predictive value for progression to severe postpartum hemorrhage of early viscoelastometric point-of-care testing for hyperfibrinolysis has been found to be limited [50].

## Choice of anaesthesia for surgical treatment of PPH

A Danish national study found that larger centres were more likely than smaller centres to use regional rather than general anaesthesia, but no evidence as to which approach was best [51].

## Pelvic artery embolization

In settings where the technology is available, observational data suggest that pelvic artery embolization is a useful treatment for PPH [52–54]. The procedure may be associated with increased risk of PPH and placenta accrete spectrum in subsequent pregnancies [55].

## Endovascular balloon occlusion of the aorta, common ileac arteries or internal ileac arteries

Resuscitative endovascular balloon occlusion of the aorta has been used for life-threatening postpartum haemorrhage, with promising results in observational studies [56]. An abdominal aortic balloon has also been placed prior to CS in cases of placenta accreta spectrum [57]. Observational studies suggest improved outcomes with balloon occlusion of the abdominal aorta, the common ileac arteries or the internal ileac arteries [58, 59]. Low quality evidence suggests that aortic occlusion may be more effective than internal ileac artery occlusion [60]. In cases of aortic balloon occlusion for obstetric haemorrhage prior to delivery, fetal/neonatal outcomes were satisfactory [61].

## External aortic compression

External aortic compression has been recommended by a WHO technical committee as part of the first line bundle of care for PPH [3]. A scoping review found that the technique is able to interrupt distal blood flow, and limited evidence of clinical effectiveness [62]. An alternative method is with the El-Minia aortic compression device comprising an abdominal belt with inflatable balloon [63].

## Direct compression of the aorta during laparotomy

In settings in which interventional radiological procedures are not available, digital occlusion or clamping of the abdominal aorta has been used, but we are not aware of any studies comparing endovascular with external occlusion procedures.

Direct digital aortic compression is an intuitive procedure for controlling postpartum haemorrhage during laparotomy which was described more than 50 years ago [64].

## Aortic clamping

A technique for infrarenal aortic clamping has been advocated for management of placenta accrete spectrum [65].

## Novel Paily aortic clamp

A novel aortic clamp which is applied without retroperitoneal dissection has been applied successfully for mean 55 (standard deviation 20) minutes in 33 cases of placenta accrete spectrum without apparent vascular complications [66].

## Transvaginal haemostatic ligation procedures

For postpartum haemorrhage arising from the lower uterine segment, traditionally managed with methods such as packing, balloons, transabdominal arterial ligation and hysterectomy, transvaginal haemostatic ligation procedures have been described [67], As well as a ‘waveform’ transverse suture with concertina effect on first the anterior then the posterior wall of the cervix and flaccid lower uterine segment has been described [68].

## Placental site sutures

A circular purse-string suture of the myometrium and serosa around the placental site has been described for controlling placental site bleeding [69].

## Uterine compression sutures

Surgical compression of the uterus with sutures is an intuitively attractive approach. Compression sutures such as the B-Lynch suture have been widely implemented and are considered effective and safe [70], despite a lack of robust evidence.

## Esike’s technique

Severeal variations on the original B-Lynch technique have been described, including Esike’s technique (three sutures anchored anteriorly and 3 posteriorly in the lower uterine segment, and tied over the fundus) [71, 72].

## Three vertical compression sutures

Another novel variation involves three vertical compression sutures, of which only the central one traverses the endometrial cavity [73].

## Vertical plus horizontal compression sutures

Another method involves bilateral vertical compression sutures of the uterine fundus plus two or three horizontal sutures of the uterine body [74].

## Parallel loop binding compression sutures

For haemorrhage from a placenta praevia site, parallel loop binding compression sutures have been described. An FG28 abdominal drainage silicone tube is passed from the uterine cavity to the vagina. The bladder is reflected inferiorly. A series of sutures encircling the cervix and lower uterine segment but excluding the descending branches of the uterine arteries are placed, about 1 cm apart, to achieve haemostasis [75].

## Uterine isthmus vertical compression sutures

A method combining an intrauterine double-balloon tamponade with a uterine isthmus vertical compression suture has been described [76]

## Isthmic circumferential suture

A circumferential isthmic compression suture was compared favourably with the Bakri balloon for treatment of uterine atony at caesarean section [77].

## Circumferential compression sutures and intrauterine balloon

This method for managing haemorrhage from placenta praevia involves no bladder reflection, a high uterotomy, insertion of a Bakri balloon or FG24 Foley balloon and placement of one or two circumferential compression sutures passing through the avascular areas of the broad ligaments around the upper and/or the lower part of the balloon [78].

## King’s combined uterine suture

For lower uterine segment haemorrhage, this procedure involves reflection of the bladder, bilateral transverse sutures through the cervix and the broad ligament to interrupt the parauterine vessels, and bilateral vertical compression sutures of the lower uterine segment [79].

## Removable retropubic uterine compression suture

A novel approach to reducing adverse long-term effects of uterine compression sutures has been described whereby the uterus is compressed against the lower abdominal wall [80].

## Innovative measures to control lower uterine segment haemorrhage associated with placenta accreta spectrum

In cases of bleeding from the anterior lower uterine segment, a novel lower uterine folding suture has been devised. The lower segment is folded by means of a purse-string suture from the upper edge of the lower segment to the internal cervical os including both anterior and lateral aspects of lower uterine segment, followed by closure of the uterine incision [81]. An alternative method is the modified cervical inversion technique [82]. Other innovations include use of a vessel sealing system to dissect the bladder from the lower uterine segment and a linear cutter to create a bloodless hysterotomy [83]. A stepwise conservative surgical approach for placenta praevia accrete involving bilateral uterine artery ligation, myometrial excision of the adherent placenta and cervico-isthmic sutures has been described [84]. The ‘triple P with T-shaped lower segment suture’ includes bilateral uterine artery ligation, excision of the myometrium with attached placenta, and reconstruction of the lower uterine segment with a T-shaped suture [85].

## ‘Sandwich’ technique

Simultaneous use of a modified B-lynch compression suture plus intrauterine balloon tamponade has been described as a novel ‘sandwich’ technique [86].

## Uterine devascularization

Traditionally, bilateral hypogastric artery ligation has been used for treatment of refractory PPH [87]. More recently, stepwise uterine devascularization has been described, with bilateral ligation of the ascending uterine arteries [88] and/or the ovarian arteries. Uterine necrosis, infection and sub-involution have been described following combined compression suture plus uterine devascularisation [89].

## Cell salvage

Cell salvage at caesarean section and vaginal delivery is constrained by concerns about infection and amniotic fluid embolism. Recent evidence suggests that this procedure may be feasible [90, 91], including at vaginal delivery [92], but the complexity of the blood preparation process may make it inaccessible in many places where donor blood products are not available.

## High frequency focussed ultrasound for placenta increta

High frequency focussed ultrasound for placenta increta has shown promising results in observational studies, including those in which the placenta is left in situ [93].

## Veno-arterial extra-corporeal membrane oxygenation (VA-ECMO)

Postpartum haemorrhage associated with amniotic fluid embolization is frequently associated with increased pulmonary artery pressures, profound hypoxia and refractory hypotension. VA-ECMO has been used to maintain circulation and oxygenation until recovery of cardiopulmonary function [94]. ECMO has also been used in postpartum refractory shock and respiratory failure, including due to PPH [95].

## Conclusions

The large number and variety of procedures described in recent years is testimony both the inventiveness of clinicians and the fact that PPH remains a major global problem with associated mortality. This review highlights the lack of robust evidence of effectiveness of most individual methods for treatment of PPH. At the same time, knowledge of the variety of innovative methods described above can equip clinicians with alternatives to implement as a last resort when faced with haemorrhage unresponsive to conventional methods, particularly in settings with limited access to resource-intensive methods.

## Data Availability

Not applicable.

## Abbreviations

CI: Confidence interval
DNI: Delta neutrophil index
HSC: Heat stable carbetocin
MAST: Medical antishock trousers
PPH: Postpartum haemorrhage
RR: Risk ratio
TVUAC: Transvaginal Uterine Artery Clamp
USD: United States Dollar
VA-ECMO: Veno-arterial extra-corporeal membrane oxygenation
WHO: World Health Organization
